# Occupancy Baselines and Temporal Trends of Threatened Mammal Communities in Southeast Asian Mangroves

**DOI:** 10.1002/ece3.74391

**Published:** 2026-09-20

**Authors:** Vanessa Herranz Muñoz, Reaksmey Sophatt, Vichet Roth, Naruemon Tantipisanuh, George Andrew Gale, José Jiménez

**Affiliations:** ^1^ Conservation Ecology Program, School of Bioresources and Technology King Mongkut's University of Technology Thonburi Bangkok Thailand; ^2^ Fishing Cat Ecological Enterprise Co. Ltd. Phnom Penh Cambodia; ^3^ Conservation Ecology Program, Pilot Plant Development and Training Institute King Mongkut's University of Technology Thonburi Bangkok Thailand; ^4^ Instituto de Investigación en Recursos Cinegéticos (IREC, CSIC‐UCLM‐JCCM) Ciudad Real Spain

**Keywords:** camera‐traps, mangroves, monitoring, occupancy modeling, Southeast Asia, threatened mammals

## Abstract

Mangroves and mangrove‐associated wetlands provide critical refuges for elusive and threatened mammals, yet long‐term population baselines are scarce. We conducted camera‐trap monitoring of mammal communities in the Koh Kapik Ramsar Site and Peam Krasop Wildlife Sanctuary, coastal Cambodia, from 2017 to 2023. We deployed cameras at 20–30 sites per year, adding up to a total of 156 sites. Using a multi‐species hierarchical occupancy framework, we estimated baseline occupancy and detection probabilities, habitat associations, anthropogenic effects, and temporal trends for eight mammal species. Endangered long‐tailed macaque ( *Macaca fascicularis* ) and Vulnerable fishing cat ( *Prionailurus viverrinus* ) showed the highest occupancy probabilities, whereas Endangered hairy‐nosed otter ( *Lutra sumatrana* ) and Critically Endangered Sunda pangolin ( *Manis javanica* ) had consistently low and uncertain occupancy. Detection probability varied markedly among species, with smooth‐coated otter ( *Lutrogale perspicillata* ) being the most detectable. Species exhibited contrasting habitat associations, with fishing cats selecting back and fringe mangroves, hairy‐nosed otters associated with riverine mixed mangroves, and large‐spotted civets ( *Viverra megaspila* ) favoring *Melaleuca* swamp forests. Occupancy increased with tree cover across the community, while responses to distance from villages differed among species, indicating both tolerance and avoidance of human activity. Temporal trend analyses suggest declines in occupancy for several threatened species, including long‐tailed macaque, fishing cat, smooth‐coated otter, and large‐spotted civet. Our results highlight the importance of mangrove‐associated habitats and forest cover for mammal persistence and provide one of the first community‐level baselines for evaluating and guiding conservation actions in Southeast Asian mangrove landscapes.

## Graphical abstract

We analyzed long‐term camera‐trap monitoring data of rare threatened mammal species in Southeast Asian mangrove habitats. We applied a multi‐species hierarchical occupancy framework to estimate baseline occupancy and detection probabilities, habitat associations, anthropogenic effects and temporal trends.

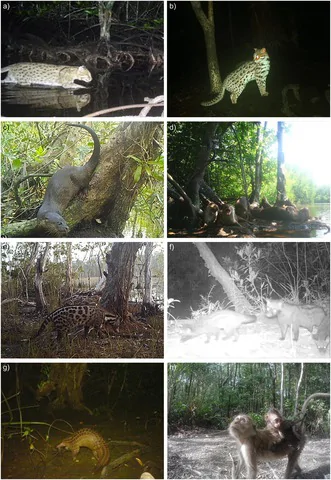

## 1 Introduction

Mangrove forests are an important refuge for rare and elusive species, particularly primate and cat species, due to their inaccessibility (Nowak 2013). Mangrove forests in mainland Southeast Asia, and particularly in Cambodia, support several threatened mammal species for which ecological information remains extremely limited: Critically Endangered Sunda pangolin, Endangered hairy‐nosed otter, large‐spotted civet, and long‐tailed macaque, Vulnerable fishing cat and smooth‐coated otter. For several of these species, rarity and cryptic behavior have resulted in a paucity of empirical data on habitat use, population status and key threats. Sunda pangolin has become extremely rare and has suffered massive declines in mainland Southeast Asia due to poaching for the wildlife trade (Challender et al. 2019). For the large‐spotted civet, habitat association remains unclear and there has been little investigation of aspects of natural history needed for conservation planning (Timmins et al. 2016; Pin, Kamler, et al. 2026; Pin, Wilting, et al. 2026). The hairy‐nosed otter was once believed to be extinct, and is considered the rarest otter in the world (Sasaki et al. 2021). Fishing cat records are particularly scarce in Southeast Asia (Mukherjee et al. 2025). In Cambodia there was only one fishing cat record until a population was identified in coastal mangroves (Thaung et al. 2018), and more recently at a second mangrove area and on the Tonle Sap Lake floodplain (Herranz Muñoz et al. 2026, Early View). Camera‐trap surveys and monitoring have proved effective in collecting data on elusive species with minimal behavioral interference (Caravaggi et al. 2017), which can be used for a great variety of ecological analyses including occupancy modeling (MacKenzie et al. 2002). Long‐term camera‐trap monitoring allows for potentially higher detection rates of elusive species and generates robust datasets for conservation planning (Bruce et al. 2025), and can also provide data to estimate temporal trends in species occupancy (Zuleger et al. 2023). Multi‐species hierarchical occupancy models combine the power of occupancy models to investigate the spatial ecology of wildlife communities, with the integration of data across species provided by hierarchical modeling, which improves abundance/occupancy estimates for rare species (Dorazio and Royle 2005). The multi‐species approach can incorporate evaluations of how habitat variables or potential threats impact the community (Russell et al. 2009). Furthermore, multi‐species models can provide community‐level occupancy baselines and associations between environmental variables, threats, and wildlife communities, including elusive species, which is essential for conservation management (Zipkin et al. 2010).

Long‐term monitoring is particularly important for the conservation of threatened species suspected to be undergoing steep declines, such as the large‐spotted civet. Long‐term monitoring data have shown a major decline of this species at one of its most important global strongholds, Srepok Wildlife Sanctuary in Eastern Cambodia, likely as a result of indiscriminate poaching. These findings highlight the need for additional monitoring in other potential strongholds to assess population status and habitat requirements (Pin, Kamler, et al. 2026; Pin, Wilting, et al. 2026). Similarly, the hairy‐nosed otter population and distribution are smaller than other Asian otter species, and anthropogenic threats such as human‐wildlife conflict, illegal wildlife trade, and loss of peat‐swamp forest habitats due to fire and urbanization have a disproportionate impact on the species (Kamjing et al. 2026).

We conducted long‐term camera‐trap monitoring of mammal species within a mangrove ecosystem in Koh Kapik Ramsar Site and Peam Krasop Wildlife Sanctuary, in coastal Cambodia, continuously between 2017 and 2023. The aim of this study was to estimate the mammal community occupancy, species occupancy, and detection baselines, and the effects of environmental and anthropogenic variables including mangrove habitat type, tree cover, habitat patch size, distance to water courses, distance to villages, and human activity. We also aimed to estimate species occupancy trends to assess potential population declines. We hypothesized that these species may use typical mangrove and mangrove‐associated habitats differently and have different associations to water courses depending on their ecology, while tree cover would have a positive effect on occupancy across the community. The effect of distance to villages and human activity was expected to vary among species, providing insight into differential tolerance to human disturbance. This information will provide baseline parameters for threatened species monitoring and to support management actions to ensure their long‐term survival. More broadly, these baselines provide a quantitative framework for evaluating future conservation interventions in mangrove landscapes of regional importance.

## 2 Methods

### 2.1 Study Area

Peam Krasop Wildlife Sanctuary and Koh Kapik Ramsar Site (11°29′24.00″N, 103°4′48.00″E) are located in southwest Cambodia, on the coastal area of Koh Kong province. Peam Krasop Wildlife Sanctuary was designated in 1993 and covers 237.5 km^2^ of evergreen and semi‐evergreen forest, as well as riverine and coastal mangroves. Koh Kapik Ramsar Site was declared in 1999 and covers 120 km^2^ of mangroves, partially overlapping with Peam Krasop Wildlife Sanctuary. Peam Krasop and Koh Kapik hold one of the largest and densest mangrove forests remaining in Southeast Asia (Marschke and Nong 2003), and provide habitat for a significant array of species including several globally threatened carnivores and primates (Heng et al. 2016; Thaung et al. 2018; Herranz Muñoz et al. 2024).

Local communities in Peam Krasop and Koh Kapik are highly dependent on natural resources. Livelihoods are mainly based around fishing, crabbing, and historically, charcoal production and exportation. Overexploitation of wildlife and nontimber forest products (NTFPs), land clearance for agriculture, land grabbing, illegal hunting, and fishing are major threats to this ecosystem (Dara et al. 2009; Kastl et al. 2013). These pressures have created a heterogeneous landscape with varying levels of human activity and accessibility, making the area particularly suitable for evaluating species‐specific responses to anthropogenic disturbance. The current study focused on the coastal and riverine mangrove and mangrove‐associated habitats of Peam Krasop and Koh Kapik covering approximately 150 km^2^. This area encompasses fringe, back, and mixed mangrove systems, as well as adjacent swamp and semi‐evergreen habitats relevant to the focal species.

#### 2.1.1 Camera‐Trapping

**FIGURE 1:**
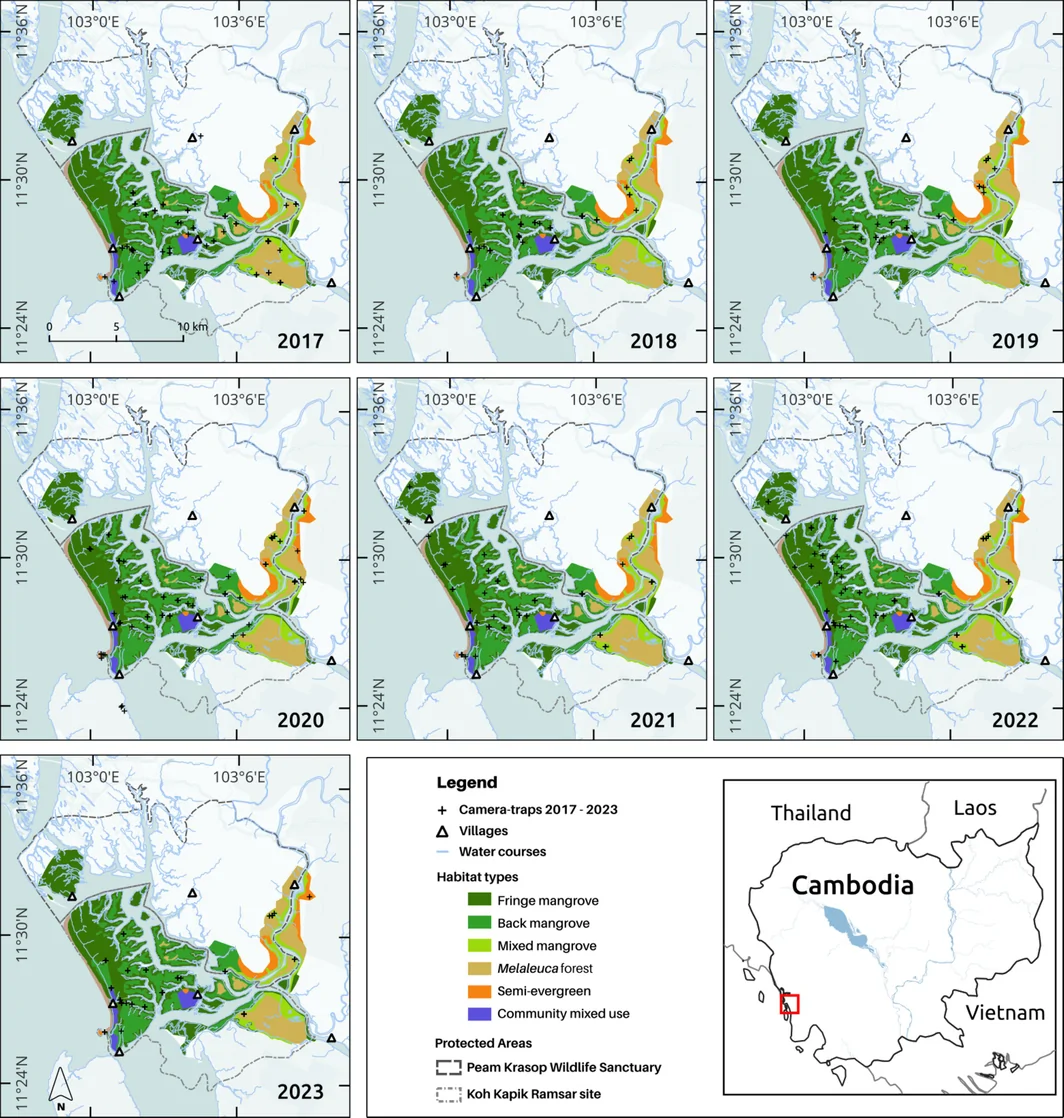
Maps of camera‐traps per year between 2017 and 2023. Habitat types as described in Table 1. Water courses, villages, and protected area boundaries.

Camera‐trap monitoring was conducted repeatedly from February 2017 to December 2023, deploying 20–30 cameras an average of 111.4 days per year. Sampling corresponded to 24‐h intervals and was used as repeated detection occasions within each site‐year unit. Across the study period, total sampling effort amounted to 26,111 camera‐trap nights, averaging 3730 (±SD = 920) camera‐trap nights per year. Deployment duration varied among cameras and years, with a median of 52 days (IQR = 31–77 days). Cameras were deployed on an approximate 1.5‐km grid, designed to cover the potential home range of an individual fishing cat, considering estimates from the literature (Cutter 2014; Adhya et al. 2024). The sampling design aimed to provide representative coverage of the study area. Cameras were deployed at a total of 156 geographically independent locations (Figure 1).

#### 2.1.2 Data Analysis

**FIGURE 2:**
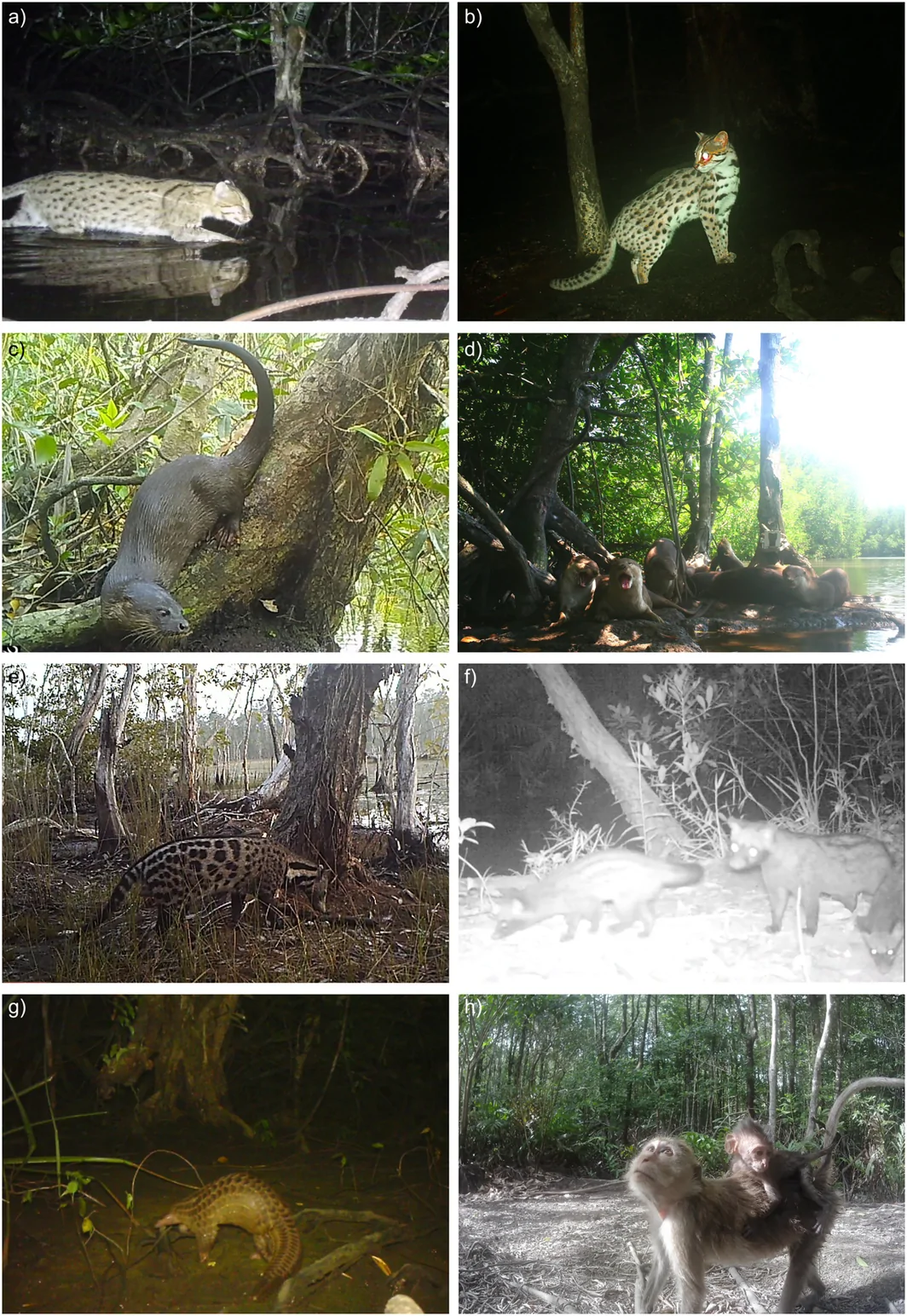
Camera‐trap photos of the study species in the mangrove habitats of Peam Krasop Wildlife Samctuary and Koh Kapik Ramsar Site: (a) fishing cat in back mangrove; (b) leopard cat in mixed mangrove; (c) hairy‐nosed otter in mixed mangrove; (d) smooth‐coated otters in fringe mangroves; (e) large‐spotted civet in *Melaleuca* forest; (f) common palm civets in semi‐evergreen forest; (g) Sunda pangolin in mixed mangrove; (h) long‐tailed macaques in fringe mangrove.

Data were compiled on eight species sharing the mangrove habitats: Sunda pangolin, hairy‐nosed otter, large‐spotted civet, long‐tailed macaque, fishing cat, smooth‐coated otter (see Figure 2), and leopard cat ( *Prionailurus bengalensis* ) and common‐palm civet ( *Paradoxurus hermaphroditus* ). The latter two more common species (categorized as Least Concern) were incorporated into the analyses to represent the full mangrove and mangrove‐associated mammal community (excluding small mammals < 1 kg), and to strengthen inference for rare species through information sharing in the hierarchical modeling framework.

Species photographic captures were compiled using 24‐h sampling occasions to produce detection/nondetection data for each camera within each year. We compiled local environmental and anthropogenic variables to explore their effect on species occupancy and detection. The variables were extracted from field data, camera‐trap data, available environmental data, or calculated in QGIS 3.10.4 (QGIS Development Team 2022), and mapped to the camera‐trap locations. Habitat types were derived from field research and ground truthing (Herranz Muñoz et al. 2024). Distance to the nearest water courses was calculated in the field and checked in QGIS through point‐to‐line distance tools to a detailed water‐line layer. Percentage of tree cover in a 25 m radius was estimated in the field. Patch size was calculated in QGIS by digitizing habitat types and measuring areas of discrete habitat type patches (Table 1).

**TABLE 1:** Environmental and anthropogenic variables incorporated in the multi‐species hierarchical models.

Model variables	Units	Description
Hab	Habitat categories	Habitat type: Types derived from field research (see Herranz Muñoz et al. 2024). Raster digitized from Sentinel‐2 2023 for the habitat categories defined. 1 = Fringe mangrove: Strips of 20–100 m of almost permanently flooded. In Koh Kapik, dominated by Rhizophora spp. and on the coastal edge by Avicennia spp. 2 = Back mangrove: intermittently flooded, inland behind the Fringe mangrove, with a higher diversity of mangrove tree species inc. *Xilocarpus* spp., *Bruguiera* spp., *Ceriops* spp., *Lumintzera* spp., 3 = Mixed mangrove: Along main rivers and small streams 1–3 km from the sea and up to > 20 km upland with *Nipa fruticans, Barringtonia* spp., *Heritiera littoralis, Pandanus* spp., *Sonneratia* spp., *Melaleuca* spp. and other semi‐evergreen. 4 = *Melaleuca* forest: Further inland from mixed mangroves, *Melaleuca* spp. dominated swamp forests 5 = Semi‐evergreen: High diversity of evergreen and deciduous species inc. *Dipteroarpus costatus, Xylia xylocarpa, Anisoptera costata and Irvingia malayana* (Rundel 1999).
DistWater	meters	Distance from the camera to nearest water source measured in the field, checked in QGIS
DistVill	meters	Distance from the camera to nearest village, produced in QGIS
DogFreq	Frequency of captures	Number of photo‐captures of dogs per 100 days at each camera location
HumanFreq	Frequency of captures	Number of photo‐captures of people per 100 days at each camera location
SmallMam	Frequency of captures	Number of photo‐captures of mammals weighing > 1 kg per 100 days at each camera location
NDVI	NDVI	Normalized Difference Vegetation Index (NDVI) points extracted in QGIS from Sentinel‐2 derived products (EU's Copernicus 2024) for each camera location and year
TreeCover	Percentage	Percentage tree cover on a 25 m radius around the camera‐traps measured in the field
PatchSize	Hectares	Size of habitat patch measured in QGIS

### 2.2 Multi‐Species Site‐Occupancy Framework

We used a hierarchical multi‐species occupancy model (MSOM; Dorazio and Royle 2005; Dorazio et al. 2006) to estimate species‐ and community‐level occupancy, detection, habitat associations, anthropogenic effects, and temporal trends across seven years of monitoring (Kéry and Royle 2021). The MSOM jointly models the latent ecological state (true occupancy) and the observation process (imperfect detection), allowing species‐specific parameters while sharing information across the community. Given insufficient data to support a fully dynamic multi‐species occupancy model, we adopted a stacked multi‐season occupancy framework (sensu Kéry and Royle 2021). This approach provided robust estimates of occupancy and detection while allowing species‐specific temporal trends to be estimated. Detailed information is provided in Appendix 1.

#### 2.2.1 Data Structure

Detection/nondetection data were organized by site, year, and species, with repeated sampling occasions aggregated within each species‐site‐year unit to define binomial detection histories. Occupancy was modeled as species‐ and year‐specific, constant within each year for a given species‐site combination, whereas detection probability varied among species and years and depended on sampling effort, which was incorporated directly into the binomial likelihood through the number of active camera‐trap days available for each site‐year unit. A full description of the 4‐D indexing, data restructuring, and justification for the vertical format is provided in Appendix 1.

#### 2.2.2 Covariates

We evaluated the effects of habitat type, distance to water, distance to villages, NDVI, tree cover, patch size, and frequencies of humans, dogs, and small mammals (as potential prey). Distances were log‐transformed and all covariates were standardized. Covariates were selected a priori based on ecological relevance, and collinearity was assessed using VIF < 3 (Zuur et al. 2009). To avoid introducing redundant pathways and potential collider bias (i.e., spurious associations arising from conditioning on a common effect), NDVI was excluded from the occupancy component, and human and dog frequencies were included only in the detection component (Stewart et al. 2023).

#### 2.2.3 Occupancy and Detection Models

Occupancy was modeled on the logit scale as a function of community‐level intercepts, species‐level random effects, year‐level random intercepts, species‐specific temporal trends (random slopes), and site–year covariates including habitat type. Tree cover was modeled as a community‐level effect, whereas distance to water and distance to villages were modeled as species‐specific effects to allow for heterogeneous responses among species. Detection followed a binomial model conditional on occupancy, with species‐ and year‐level random effects and sampling effort as a covariate. Model formulations for both sub‐models are provided in Appendix 1.

#### 2.2.4 Model Selection and Implementation

Candidate models were specified a priori to represent alternative biologically motivated hypotheses regarding environmental and anthropogenic effects on occupancy and detection, rather than a sequential model‐building procedure. We compared alternative model structures using Pareto‐smoothed importance sampling leave‐one‐out cross‐validation (PSIS‐LOO; Vehtari et al. 2017, 2024), marginalizing latent occupancy indicators to ensure stable predictive densities. The final model was selected as the most parsimonious structure whose leave‐one‐out expected log predictive density (LOO‐ELPD) was statistically indistinguishable from the top‐ranked model while retaining ecologically justified parameters. The final model was implemented in JAGS (Plummer 2003) via jagsUI (Kellner 2020) in R (R Core Team 2026). We ran three MCMC chains for 1,000,000 iterations, with a burn‐in of 50,000, and thinning of 10, resulting 285,000 posterior samples per parameter. Convergence was assessed via visual inspection and $\overset{`}{R}<1.1$ (Brooks and Gelman 1998). Full JAGS code and data are provided in Zenodo repository (https://doi.org/10.5281/zenodo.19679361).

## 3 Results

Monitoring data between 2017 and 2023 on the eight selected species added up to 26,111 camera‐trap nights, with an average of 3730 (±SD = 920) camera‐trap nights per year. Across all candidate models, Pareto‐k diagnostics identified a small number of observations with *k* > 0.7, indicating some influential observations, but no observations exceeded *k* = 1.0, the range in which the PSIS‐LOO approximation is generally considered unreliable (Vehtari et al. 2024). Therefore, exact leave‐one‐out refitting (*reloo*) was not required. Model selection strongly supported models where occupancy depended on habitat type, tree cover, and species‐specific responses to distance to villages and water sources. Including dog and human frequency as detection covariates did not improve model predictive performance, and detection was adequately described by species‐ and year‐level random effects without additional covariates. Patch size received little support once other habitat and anthropogenic gradients were included (Table 2).

**TABLE 2:** Comparison of alternative multi‐species occupancy models using leave‐one‐out cross‐validation (LOO).

Model	Looic	Δlooic	SE Δlooic	Rank
ψ[TreeCover, DistVill[sp], DistWat[sp], Hab] p[.]	4737.70	0.00	0.00	1
ψ [TreeCover, DistVill[sp], DistWat[sp], Hab] p [HumanFreq]	4743.59	5.88	5.94	2
ψ [DistVill[sp], DistWat[sp], Hab] p [.]	4744.05	6.35	5.52	3
ψ [TreeCover, DistVill[sp], DistWat[sp], PatchSize, Hab] p [HumanFreq]	4745.05	7.35	5.98	4
ψ [TreeCover, DistVill[sp], Hab] p [HumanFreq]	4799.03	61.33	15.14	5
ψ [Hab] *p*[.]	4858.45	120.75	22.00	6
ψ [TreeCover, Hab] p [HumanFreq]	4860.36	122.66	22.51	7
ψ [.] p [.]	4894.80	157.10	25.61	8

*Note:* For each candidate model, the table reports the LOO information criterion (looic), its difference relative to the best‐supported model (Δlooic), the standard error of this difference (SE Δlooic), and the resulting model rank. Models vary in the set of covariates included in the occupancy component (ψ) and the detection component (p). Species‐specific slopes are indicated by [sp]. Lower looic values indicate better out‐of‐sample predictive performance. Model comparison was conducted among a predefined set of plausible candidate models (see Methods). Following the LOO‐based model‐selection criterion used in the study, models with Δlooic within one standard error of the minimum were considered statistically indistinguishable; among these, the most parsimonious model was selected as the final model. All candidate models shared the same underlying random‐effects structure (species‐ and year‐level random effects), and differed only in the inclusion of fixed effects (covariates) in the occupancy (ψ) and detection (p) components. Species‐specific slopes are indicated by [sp].

Occupancy estimates (ψ) varied widely across species (Table 3). Long‐tailed macaque showed the highest occupancy, whereas fishing cat, common palm civet, smooth‐coated otter and leopard cat exhibited intermediate values. Large spotted civet and hairy‐nosed otter had low occupancy, and Sunda pangolin showed the lowest and most uncertain estimates, consistent with its very limited detections. Detection probabilities (p) also varied markedly among species (Table 3). Smooth coated otter showed by far the highest detectability, whereas most species had low to very low detection probabilities, including fishing cat and Sunda pangolin, which exhibited the lowest and most uncertain estimates.

**TABLE 3:** Posterior summaries of species occupancy (ψ) and detection (*p*), including the medians, standard deviations (SD), and 95% credible intervals. Species are ordered from highest to lowest occupancy. Sampling was conducted over 24‐h occasions.

Common name	Species	Sites detected	Median ψ	SD	95% CI	Median *p*	SD	95% CI
Long‐tailed macaque	*Macaca fascicularis*	101	0.606	0.028	0.550–0.661	0.071	0.002	0.067–0.076
Fishing cat	*Prionailurus viverrinus*	25	0.272	0.050	0.193–0.387	0.013	0.002	0.009–0.018
Common palm civet	*Paradoxurus hermaphroditus*	41	0.258	0.029	0.205–0.318	0.030	0.000	0.027–0.039
Smooth‐coated otter	*Lutrogale perspicillata*	32	0.236	0.021	0.196–0.279	0.150	0.010	0.139–0.160
Leopard cat	*Prionailurus bengalensis*	20	0.153	0.030	0.105–0.221	0.020	0.004	0.013–0.028
Large‐spotted civet	*Viverra megaspila*	10	0.081	0.022	0.048–0.134	0.021	0.006	0.011–0.034
Hairy‐nosed otter	*Lutra sumatrana*	4	0.061	0.013	0.038–0.089	0.065	0.008	0.050–0.082
Sunda pangolin	*Manis javanica*	2	0.045	0.106	0.008–0.412	0.005	0.007	0.000–0.027

**FIGURE 3:**
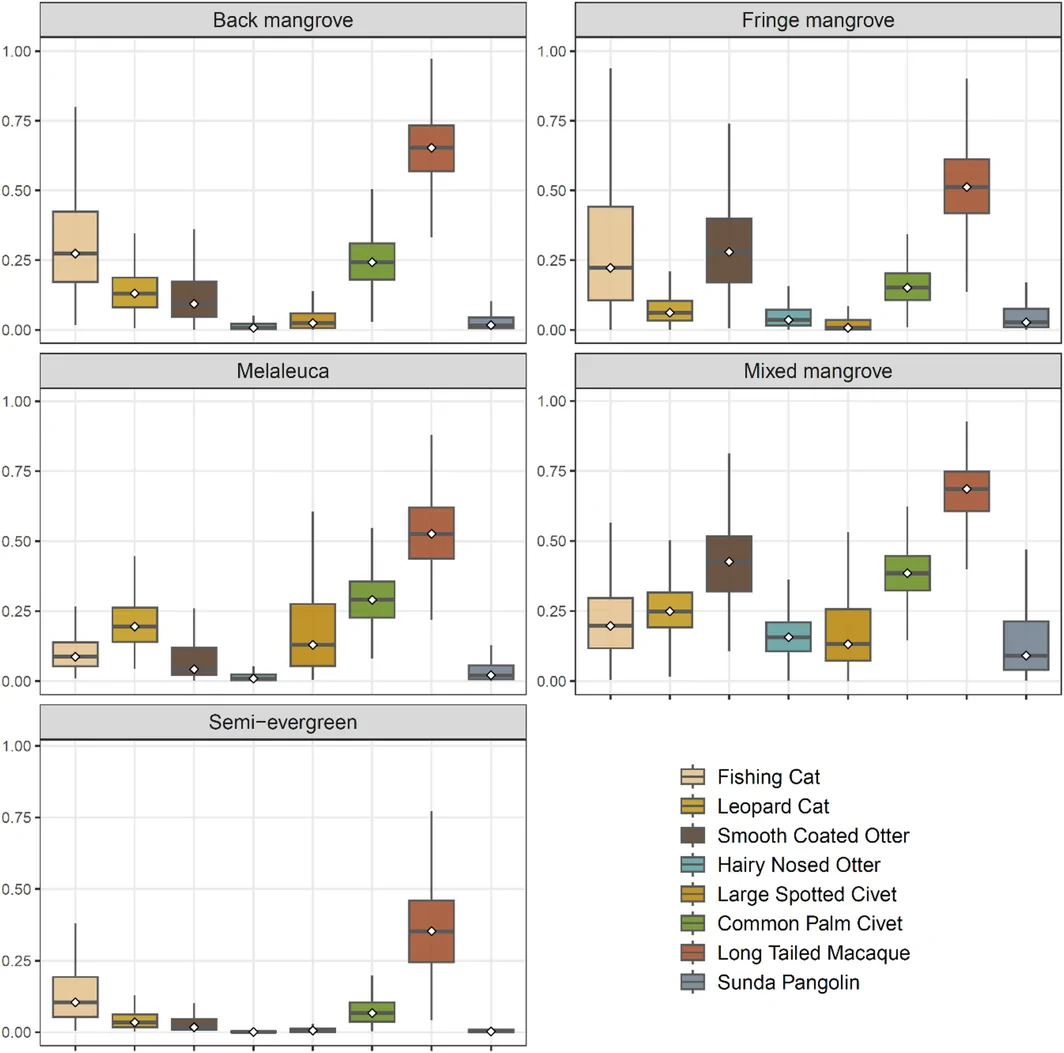
Species occupancy (*ψ*) across habitat types. Each panel corresponds to one habitat category, and boxplots summarize the posterior distribution of occupancy for each species within that habitat.

Occupancy estimates varied considerably across habitats for most species (Figure 3). Long‐tailed macaque consistently showed the highest occupancy across all habitat types, with especially elevated values in back‐ and mixed‐mangrove habitats. Several carnivores, including fishing cat, smooth‐coated otter, and common palm civet, also exhibited moderate occupancy in mangrove habitats, though often with wide credible intervals indicating substantial uncertainty. In contrast, hairy‐nosed otter, large spotted civet, and Sunda pangolin showed uniformly low occupancy across habitats, with values frequently approaching zero in semi‐evergreen forest and back‐mangrove areas. Leopard cat displayed intermediate occupancy but with clear differences among habitats, showing higher use of *Melaleuca* and mixed‐mangrove habitats. Overall, the results reveal strong habitat‐specific patterns in site use, with some species exhibiting broad habitat associations and others showing marked restrictions or very low probabilities of occurrence across most habitats.

**FIGURE 4:**
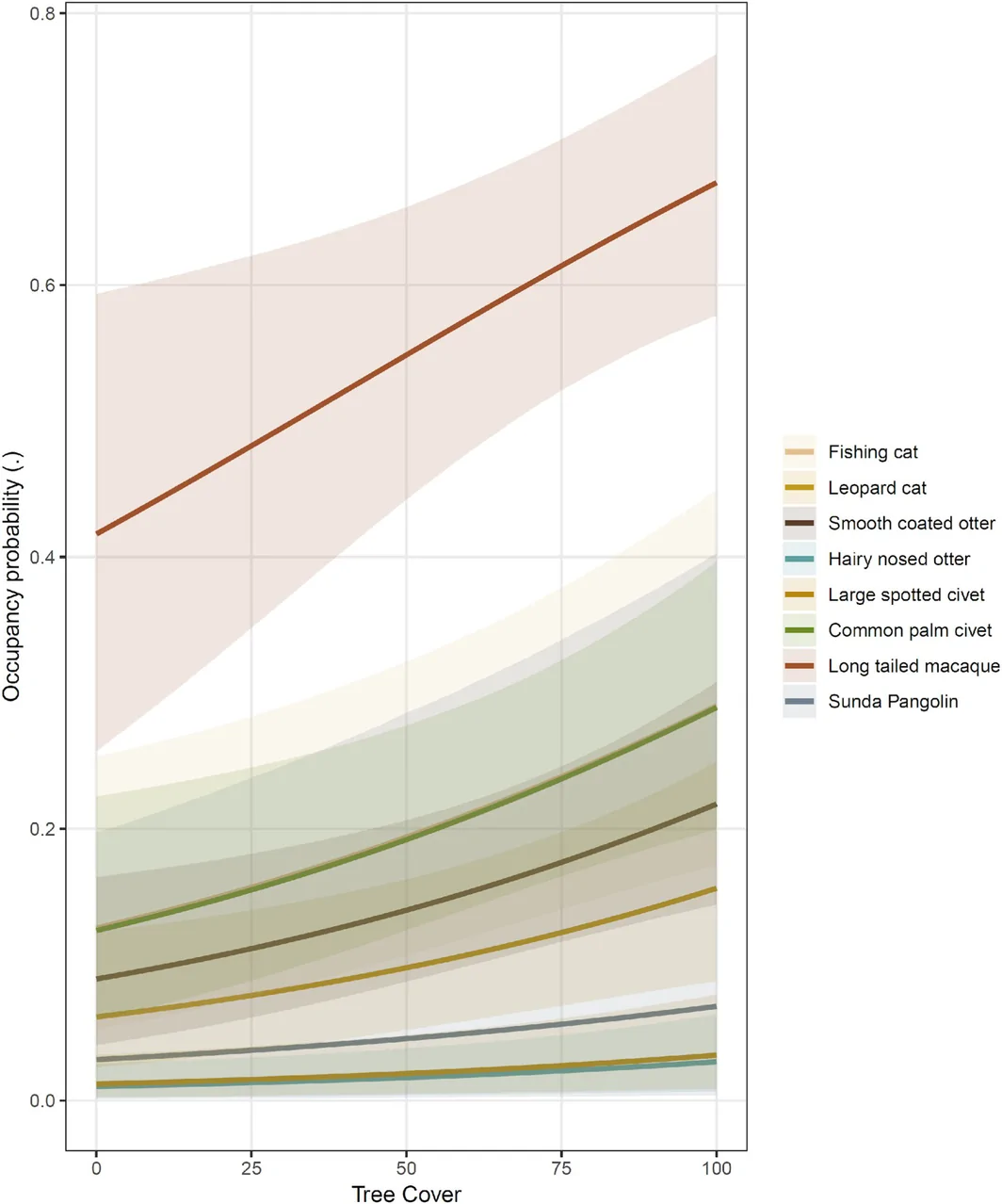
Species‐specific marginal relationships between occupancy probability (ψ) and tree cover. Curves represent posterior mean estimates of ψ across the observed range of tree cover, with shaded bands indicating 95% credible intervals. Responses are derived from a model with species‐specific intercepts and a common community‐level effect of tree cover. Differences among species reflect variation in baseline occupancies. All other covariates are held at their reference or mean values.

**FIGURE 5:**
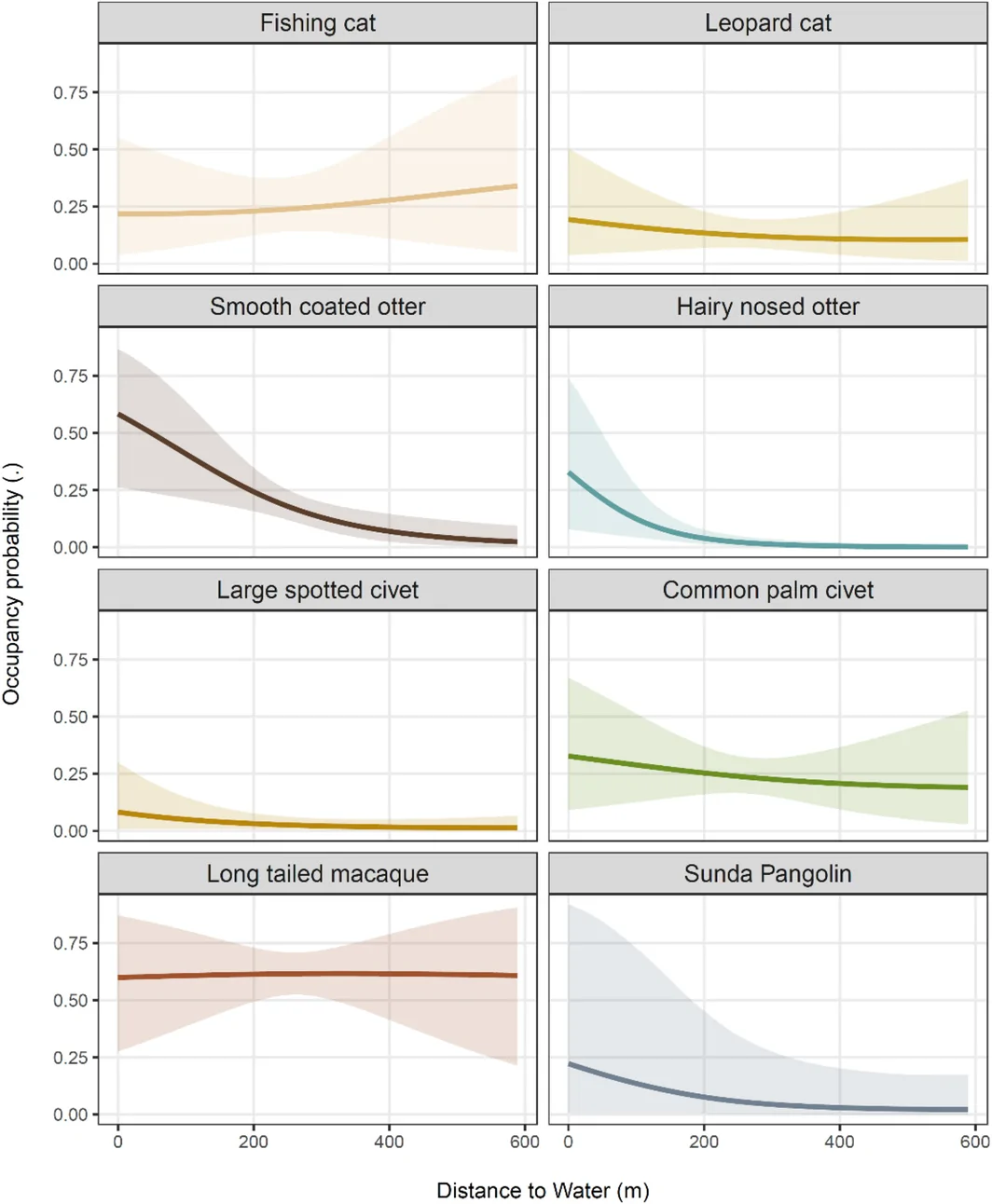
Species‐specific marginal occupancy responses to distance to water. Curves show the posterior mean occupancy probability (ψ) across the observed range of the covariate, with shaded bands representing 95% credible intervals. Panels correspond to individual species, illustrating contrasting sensitivities to proximity to water sources.

All species showed increasing probability of occupancy with higher tree cover (Table 4; Figure 4). The models also indicated species‐specific effects for distance to water, which showed that the occupancy probability of the two otter species, large‐spotted civet and Sunda pangolin, declined with increasing distance, whereas the probability of occupancy increased slightly with distance to water for fishing cat and did not show a marked effect for leopard cat, long‐tailed macaque, or common‐palm civet (Figure 5).

**TABLE 4:** Posterior summaries of occupancy (ψ) covariate effects from the multispecies occupancy model.

Parameters	Mean ψ	SD	Q2.5%	Q97.5%
**b_treecover**	**0.204**	**0.077**	**0.053**	**0.355**
beta_DistWater [Fishing cat]	0.498	0.297	−0.019	1.153
beta_DistWater [Leopard cat]	0.081	0.227	−0.369	0.526
**beta_DistWater [Smooth‐coated otter]**	**−0.922**	**0.194**	**−1.315**	**−0.554**
**beta_DistWater [Hairy‐nosed otter]**	**−1.673**	**0.561**	**−2.934**	**−0.735**
beta_DistWater [Large‐spotted civet]	−0.309	0.330	−0.986	0.315
beta_DistWater [Common palm civet]	0.080	0.173	−0.259	0.421
**beta_DistWater [Long‐tailed macaque]**	**0.334**	**0.152**	**0.041**	**0.637**
beta_DistWater [Sunda pangolin]	−0.809	0.739	−2.476	0.379
**beta_DistVill [Fishing cat]**	**−1.178**	**0.308**	**−1.878**	**−0.666**
**beta_DistVill [Leopard cat]**	**0.579**	**0.279**	**0.061**	**1.156**
**beta_DistVill [Smooth‐coated otter]**	**−0.444**	**0.150**	**−0.739**	**−0.152**
beta_DistVill [Hairy‐nosed otter]	0.426	0.315	−0.157	1.079
**beta_DistVill [Large‐spotted civet]**	**1.920**	**0.579**	**0.903**	**3.170**
beta_DistVill [Common palm civet]	0.270	0.182	−0.077	0.637
**beta_DistVill [Long‐tailed macaque]**	**−0.339**	**0.141**	**−0.621**	**−0.067**
beta_DistVill [Sunda pangolin]	0.315	0.733	−0.974	1.869

*Note:* The table reports posterior means, standard deviations, and 95% credible intervals for the community‐level effect of tree cover and species‐specific effects of distance to water and distance to villages. Parameters in bold indicate effects for which 95% credible intervals do not overlap zero.

**FIGURE 6:**
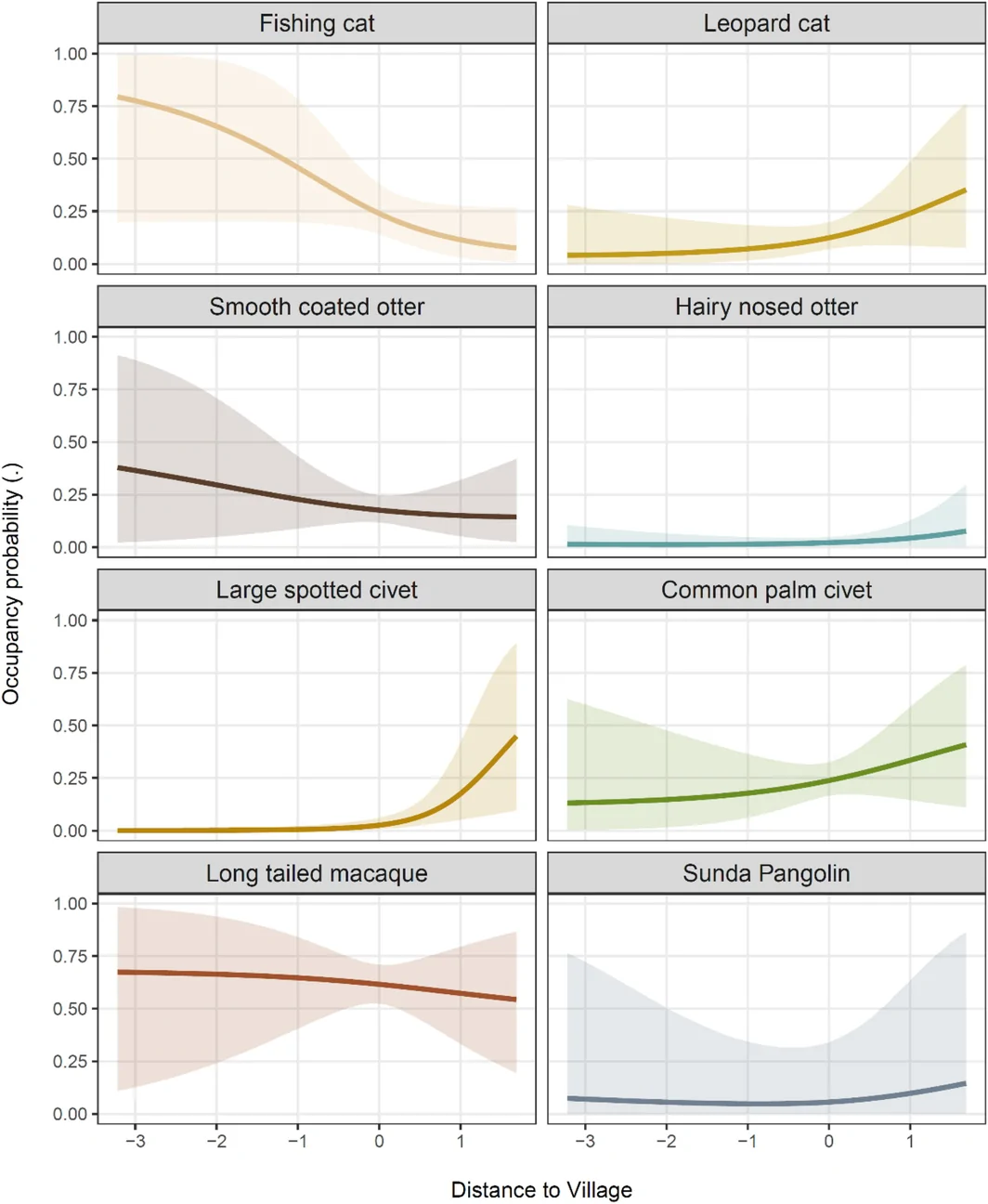
Species‐specific marginal occupancy responses to distance to villages. Curves represent the posterior mean occupancy probability (ψ) across the observed range of the standardized covariate, with shaded bands indicating 95% credible intervals. Panels correspond to individual species, highlighting contrasting sensitivities to human proximity.

**FIGURE 7:**
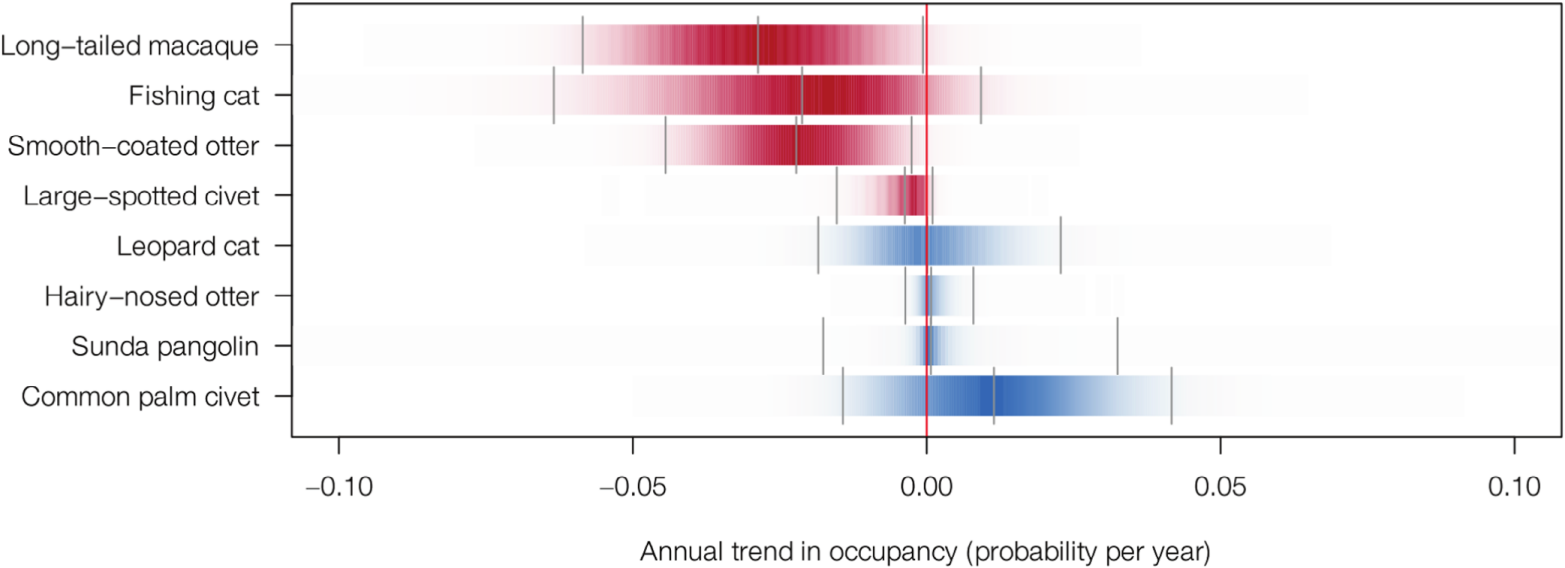
Annual trends in occupancy probability per year (posterior mean *gamma*), mean and 95% CI for each species. Red denotes a negative slope, blue denotes slope near 0 or positive.

The effect of distance to the nearest village also suggested species‐specific effects and showed that fishing cat, long‐tailed macaque, and smooth‐coated otter occupancy tended to be higher near villages, while the probability of occupancy increased further away from villages for large‐spotted civet, common‐palm civet, leopard cat, Sunda pangolin, and hairy‐nosed otter (Figure 6). Modeling species‐specific temporal trends, incorporated as random slopes using a centered year covariate, showed that long‐tailed macaque, fishing cat, smooth‐coated otter, and large‐spotted civet probability of occupancy declined over the survey period. Leopard cat, hairy‐nosed otter, and Sunda pangolin probability of occupancy remained stable, whereas common‐palm civet occupancy increased (Figure 7).

## 4 Discussion

Our analyses estimated values for occupancy and detection for eight species of interest in the mangrove forests of Peam Krasop Wildlife Sanctuary and Koh Kapik Ramsar site, providing a suitable baseline for species monitoring. Occupancy and detection probabilities are often highly site specific and dependent on the species targeted by the survey (Shannon et al. 2014). In broad terms, our results reflect low to very low (< 0.3) parameters for all species except long‐tailed macaques (Table 3). Fishing cat occupancy was similar to probabilities obtained in human‐dominated landscapes in Thailand and considered to reflect a range of pressures (Hohlfeld et al. 2026). Smooth‐coated otter occupancy was close to the lower end of ranges reported in other sites in the region (e.g., Tantipisanuh et al. 2023), however accurate study of otter species occupancy will require targeted surveys.

Species exhibited clear differences in their associations with habitat (Figure 3), reflecting contrasting patterns of site use across the mangrove landscape. Semi‐aquatic species such as both otters showed higher occupancy probabilities closer to water sources, although their habitat associations differed, with smooth‐coated otters using both fringe and mixed mangroves, whereas hairy‐nosed otters were more strongly associated with riverine mixed mangrove habitats. In contrast, fishing cats and long‐tailed macaques were more likely to occur in back‐mangrove areas, not necessarily in close proximity to water. Large‐spotted civets showed the strongest association with *Melaleuca* swamp forests, suggesting a more restricted pattern of habitat use.

Across the community, occupancy probability tended to increase with tree cover, indicating that areas with greater forest structure may provide more suitable conditions for multiple species. While these patterns are correlational, they are consistent with the importance of structurally complex mangrove and mangrove‐associated habitats for supporting mammal communities in this ecosystem (e.g., Pardo et al. 2024). Some studies have found potential thresholds of forest cover loss, beyond which populations can decline rapidly (e.g., Roque et al. 2018), suggesting that this community may be particularly sensitive to reductions in forest cover.

Anthropogenic effects were assessed by testing distance to villages as an occupancy covariate and human and dog frequency as detection covariates. Model selection retained distance to villages, while human and dog frequency were not supported in the detection component of the final model. Fishing cats, smooth‐coated otters and long‐tailed macaques were more likely to occupy areas closer to villages. This pattern suggests spatial tolerance to human proximity and a degree of coexistence within human‐modified landscapes. Effects were generally minor and presented large CIs, indicating tolerance rather than attraction, as well as indicating that villages are not geographical sources of threats. Fishing cats have been found to make extensive use of anthropogenic landscapes (e.g., Mishra et al. 2021; Adhya et al. 2024; Phosri et al. 2021, 2025), smooth‐coated otters are considered to be exceptionally resilient in the presence of human activity (Theng and Sivasothi 2016; Khoo and Sivasothi 2018), and long‐tailed macaques are widely known to behave as opportunists in human‐modified habitats (Gumert 2011; Marty et al. 2020). Conversely, hairy‐nosed otter, large‐spotted civet and Sunda pangolin occupancy probability increased further away from villages. Effects were also weak and had large CIs, reflecting overall rarity of these species within the sampled areas: further research should use habitat association results to target surveys for these species. For the large‐spotted civet, our results support previous findings that records generally come from large blocks of undisturbed natural habitats (e.g., Holden and Neang 2009; Jenks et al. 2010; Chutipong et al. 2014; Pin, Kamler, et al. 2026). The hairy‐nosed otter has been found by a few studies to inhabit disturbed secondary forests (Heng et al. 2016) or human‐modified landscapes such as oil palm plantations and mining ponds (Abdul‐Patah et al. 2020), however our findings support preferential selection of areas away from human centres of activity, as it has been shown in Bor Lor and Thale Noi Non‐Hunting Areas in Thailand (Kamjing et al. 2026).

Analyses of trends over the 7 years of monitoring showed declines in occupancy for the Endangered long‐tailed macaque and large‐spotted civet, and Vulnerable fishing cat and smooth‐coated otter. These results fit with the regional trends detected for some of these species during the same period, exemplified by the reassessments of long‐tailed macaques from Least Concern to Vulnerable in 2020, to Endangered in 2022 (Hansen et al. 2025). Conversely, the species‐specific temporal slope was positive for one of the generalist species, the common palm civet. Generalist species are more widespread and adaptable to a variety of habitats (Duckworth et al. 2024). Their increase in occupancy within mangroves, taken together with the decline in occupancy of fishing cats detected, may possibly reflect meso‐predator release (e.g., Ritchie and Johnson 2009). The smaller carnivores may have increased their area of occupancy with the decline in occupancy of the larger fishing cats, which may have otherwise occasionally killed or predated on them (e.g., Palomares and Caro 1999), or kept them away out of fear (Laundre et al. 2001).

Our study demonstrates the importance of long‐term monitoring to make ecological and conservation inferences for rare and elusive threatened species in mangroves and wetlands. Furthermore, it is also essential to extend surveys to other areas of suitable habitats highlighted in this study in order to ensure protection of their fragmented populations, as shown by new records of fishing cat in other Cambodian mangrove areas and the Tonle Sap floodplain, and large‐spotted civet and hairy‐nosed otter in riverine mixed mangroves and *Melaleuca* forests in Sre Ambel district (Pin, Kamler, et al. 2026; Pin, Wilting, et al. 2026; Herranz Muñoz et al. 2026, Early View; Fishing Cat Ecological Enterprise 2026).

### 4.1 Conservation Implications

Our results show that the mangrove mammal community of Koh Kapik Ramsar Site and Peam Krasop Wildlife Sanctuary is likely to be sensitive to tree cover loss and loss of habitat types. Threatened species showed clear preferential use of different mangrove and mangrove‐associated habitats, highlighting the importance of protecting not only typical mangrove forest but also associated habitats: *Melaleuca*‐dominated swamp forests and riverine mixed mangroves.

Our analyses do not provide enough information to ascribe the decline in occupancy to specific causes or processes. However, the study period overlapped the COVID‐19 pandemic, when deforestation and poaching in Cambodia and other tropical countries increased due to reverse migration (Davis et al. 2023; Singhal et al. 2024). Indiscriminate poaching, particularly using snares, continues to be an urgent and pressing threat to wildlife in Cambodia and Southeast Asia (Gray et al. 2017).

Although dog frequency was not retained in the final model, dogs may nonetheless pose direct and indirect risks to the focal species, as is the case for fishing cats in Nepal and Thailand (e.g., Mishra et al. 2021; Phosri et al. 2021). Smooth‐coated otters and hairy‐nosed otters, as well as other carnivore species are also at risk of disease transmission from dogs (Wipf et al. 2025). In the study area, most dogs were not completely feral, they roamed from human habitations or were taken to more remote areas by people on boat, and may be used for poaching as in other parts of the country (Ibbett et al. 2021). During the study period (2017–2023) there were reports of an otter cub and a fishing cat killed in confrontations with local dogs. At the same time, our results suggest a pattern of spatial coexistence between local communities and wildlife. However, inference from these results is limited to spatial variation in occupancy probability. Because occupancy reflects patterns of site use, it cannot be interpreted as evidence of coexistence in demographic terms, nor as an indicator of reduced hunting pressure or population viability. Consequently, the potential impacts of dogs and poaching represent a conservation concern that is not captured by occupancy patterns alone but may nevertheless influence population persistence.

In this context, conservation actions to ensure the persistence of the mammal community and particularly the more sensitive threatened species hinge on effective habitat protection and restoration, and protection from poaching. Promoting responsible dog ownership, and particularly limiting their access to mangrove forests would also benefit the conservation of these species. Protected areas in Cambodia need additional financial and technical support to implement effective conservation actions (Souter et al. 2016). However, effective conservation can be enhanced by engaging local Community‐based Organizations (CBOs) in conservation activities including patrolling, in collaboration with environmental authorities (Struebig et al. 2025). In addition, supporting sustainable local livelihoods and community‐led conservation, including raising awareness to mitigate indiscriminate poaching, may be most effective (Green et al. 2023). Promoting a shift in local livelihoods towards conservation activities such as mangrove and wetland restoration, habitat monitoring, community‐based ecotourism or other nature‐based solutions may further enhance the local perception of the diverse values of nature and promote local guardianship (Pascual et al. 2023). Pressure on natural resources and potential incentives for illegal activities such as logging and poaching may also be reduced by innovative systems such as a Conservation Basic Income funded through payments for ecosystem services, carbon credits, development or other sustainable sources (de Lange et al. 2023). In addition, a sustainable finance framework for habitat protection and implementation of conservation and livelihood actions by authorities, communities and other stakeholders, such as a REDD+ or Blue Carbon initiative could be highly beneficial for habitats and species in Koh Kapik Ramsar Site and Peam Krasop Wildlife Sanctuary.

Overall, our findings highlight the importance of structurally complex mangrove and mangrove‐associated habitats for supporting mammal communities, while also providing a quantitative baseline to monitor future changes in species' site use. Follow up monitoring should be conducted annually to provide comparable data for further assessments. These results offer a foundation for evaluating and guiding conservation actions, including landscape‐scale initiatives such as REDD+ or Blue Carbon projects in rapidly changing coastal ecosystems.

## Author Contributions

**Vanessa Herranz Muñoz:** conceptualization (lead), data curation (equal), formal analysis (equal), funding acquisition (lead), investigation (lead), methodology (equal), visualization (equal), writing – original draft (lead). **Reaksmey Sophatt:** investigation (equal), methodology (equal), writing – review and editing (supporting). **Vichet Roth:** investigation (equal), methodology (equal), writing – review and editing (supporting). **Naruemon Tantipisanuh:** writing – review and editing (equal). **George Andrew Gale:** conceptualization (equal), supervision (lead), writing – original draft (equal). **José Jiménez:** data curation (equal), formal analysis (lead), methodology (equal), visualization (equal), writing – original draft (equal).

## Funding

This work was supported by Petchra Pra Jom Klao Ph.D. Research Scholarship, Fauna and Flora International, Mohamed bin Zayed Species Conservation Fund, Critical Ecosystem Partnership Fund, Fishing Cat Conservation Alliance, and Panthera.

## Ethics Statement

Research permission was granted by the Royal Government of Cambodia, and abided by the Ecology and Evolution guidelines on ethical standards. Camera‐trap images of people were used only for calculating human frequency, were stored securely to ensure their privacy, and neither have been nor will be used for any other purpose.

## Conflicts of Interest

The authors declare no conflicts of interest.

## Data Availability

Data and code are publicly available in the Zenodo repository at https://doi.org/10.5281/zenodo.19679361.

## Appendix 1

### Model Specification

We implemented a hierarchical multi‐species site‐occupancy model (MSOM) comprising two linked components: an ecological sub‐model describing the latent occupancy state and an observation sub‐model describing imperfect detection. Both components included community‐level parameters and species‐specific random effects, enabling partial pooling across the community while retaining species‐level inference.

Occupancy was modeled as species‐ and year‐specific, with a community‐level intercept, species‐level random intercepts, year‐level random intercepts, species‐specific temporal trends (random slopes), and site–year covariates retained in the final model (habitat type, tree cover, distance to water, and distance to villages). Detection was modeled conditional on occupancy using a binomial formulation with species‐ and year‐level random effects. The number of sampling occasions per site–year combination was incorporated directly through the binomial likelihood.

All continuous covariates were standardized, and distances were log‐transformed. Occupancy and detection intercepts were assigned $\text{Beta}\left(1,1\right)$ priors on the probability scale. Regression coefficients were assigned weakly informative logistic priors centered on zero, and standard deviation parameters were assigned $\text{Uniform}\left(0,5\right)$ priors to promote partial pooling while allowing substantial interspecific heterogeneity.

#### Data Structure and Indexing

Detection/nondetection data were conceptually organized in a four‐dimensional structure defined by site $\left[i\right]$, year $\left[t\right]$, sampling occasion $\left[j\right]$, and species $\left[k\right]$, with sampling occasions aggregated within each site–year–species unit. For each site–year combination, photographic records were aggregated into 24‐h sampling occasions, which served as temporal replicates. For model implementation, the hierarchical 4‐D structure $\left[i,t,j,k\right]$ was collapsed into a single indexed dimension s, where each element represents a unique site–year–species combination, yielding a vertical data structure suitable for joint modeling of ecological and observational processes. Under this indexing:

- $i\left[s\right]$ identifies the site associated with row s,
- $t\left[s\right]$ identifies the year,
- $k\left[s\right]$ identifies the species,
- and the number of sampling occasions for that unit is stored as nOccs.

Detection histories within each site–year–species unit were summarized as the number of detections $y_{s}$ out of nOccs occasions, consistent with a binomial detection model. This restructuring preserves the hierarchical relationships among sites, years, and species while enabling efficient computation and clear alignment between the latent occupancy state $z_{s}$ and the detection model.

All site‐level and site–year covariates were merged into the vertical structure, ensuring that each row contains the complete set of predictors required for the occupancy and detection sub‐models.

#### Occupancy Sub‐Model

For each species *k*, site *i*, and year *t*, the latent occupancy state is:

$$\text{logit}\left(\psi_{s}\right)=\mu_{\text{lpsi}}+\epsilon_{\psi ,k\left[s\right]}+a_{t\left[s\right]}+\gamma_{k\left[s\right]}\cdot {\text{year}}_{t\left[s\right]}+\sum_{m}\beta_{m,k\left[s\right]}X_{m,s}+\alpha_{\mathrm{hab}\left[s\right]}$$

where

$$\sum_{m}\beta_{m,k\left[s\right]}X_{m,s}=\beta_{\mathrm{TC}}\text{TreeCove}r_{s}+\beta_{\mathrm{DW},k\left[s\right]}\text{DistWa}t_{s}+\beta_{\mathrm{DV},k\left[s\right]}\text{DistVil}l_{s}$$

- $\mu_{\text{lpsi}}$ is the community‐wide intercept, representing the average occupancy across species, sites, and years,
- $\epsilon_{\psi ,k}∼N\left(0,\sigma_{\psi }^{2}\right)$ is the species–level random effects, allowing each species to deviate from the community mean,
- $a_{t}∼N\left(0,\sigma_{\text{year}}^{2}\right)$ is the year‐level random intercepts, capturing interannual variation shared across species,
- $\gamma_{k}∼N\left(0,\sigma_{\gamma }^{2}\right)$ is the species‐specific temporal trend, modeling directional changes in occupancy through time,
- $\mathrm{yea}r_{t}$ is a centered numeric index of sampling year,
- $\beta_{\mathrm{TC}}$ is the community‐level slope for tree cover, shared across all species,
- $\beta_{\mathrm{DW},k}$ is the species‐specific slope for distance to water (log‐transformed and standardized),
- $\beta_{\mathrm{DV},k}$ is the species‐specific slope for distance to villages (log‐transformed and standardized),
- $\alpha_{\mathrm{hab}\left[s\right]}$ is the habitat‐type effect, modeled as a categorical fixed effect.

#### Detection Sub‐Model

Conditional on occupancy, detection for each indexed unit s followed a binomial model:

$$y_{s}∼\text{Binomial}\left(p_{s}\cdot z_{s},\text{nOccs}\right)$$

Detection probability was modeled on the logit scale as follows:

$$\text{logit}\left(p_{s}\right)=\mu_{\mathrm{lp}}+\epsilon_{p,k\left[s\right]}+b_{t\left[s\right]}$$

where $y_{s}$ is the number of detections, $p_{s}$ is the per‐occasion detection probability, $z_{s}$ is the latent occupancy state, nOccs is the number of sampling occasions, $\mu_{\mathrm{lp}}$ is the community‐level intercept, $\epsilon_{p,k\left[s\right]}∼N\left(0,\sigma_{p}^{2}\right)$ are species‐level random effects, $b_{t\left[s\right]}∼N$ are year‐level random effects.
